# Bibliography

**Published:** 1904-10-15

**Authors:** 


### BIBLIOGRAPHY.

W. B. Saunders & Co., of Philadelphia, have just issued the second edition of the Bohm, Davidoff, Huber Text-book of Histology. It is a handsomely made volume of over 500 pages and has been thoroughly revised and brought down to date, with much new matter. As a convenience for laboratory work it is bound in flexible covers. This is one of the most popular text-books on histology in use on the Continent, and the fact that a new edition is so soon called for indicates that the Huber translation is meeting the approval of the English and American teachers. The text is clear and logically arranged, beginning with the latest technical methods, and then every tissue and organ in the human body, considering comprehensively yet without unessential details which are more or less confusing to students. The teeth and oral structures have received more satisfactory treatment than can be found in any other general work on this subject. In fact there does not seem to be a noticeable over-attention to any one subject at the expense of another, as is too frequently the case in the text-books from our scientific investigators. This work, coming as it does, fresh from the workshops of three such indefatigable students and teachers as Drs. Bohm, Davidoff and Huber, ought to command the confidence of all teachers and students and meet with much favor. We most cordially commend it to our dental instructors.

